# 

**Published:** 1790

**Authors:** 


### CONTENTS.

Page

I. *AN Account of uncommon Symptoms succeeding the Measles; with some additional Remarks on the Infection of Measles and Small Pox. By Mr.* James Lucas, *one of the Surgeons of the General Infirmary at Leeds.* 325

II. *Observations on the Angustùra Bark. By Mr.* G. Wilkinson, *Surgeon at Sunderland, Member of the Royal College of Surgeons, and honorary Member of the Chirurgo-physical Society of Edinburgh.* —— 331

III. *A Case of monstrous Birth. By Mr.* Richard Dinmore, *Surgeon at Watton in Norfolk.* —— —— 339

IV. *Case of a scirrhous Affection of the Stomach; with an Account of the Appearances on Dissection. By* Robert Graves, *M. D. Physician at Sherborne in Dorsetshire, and Extra-Licentiate of the College of Physicians, London.* —— —— 343

V. *Some Reflections on the Paracentesis of the urinary Bladder; with a Description of an Instrument employed in puncturing the Bladder through the Rectum. By Mr.* Henry Watson, *F. R. S. Surgeon to the Westminster Hospital.* —— —— 349

VI. *Account of a Wound of the ulnar Artery, at the Wrist, cured by tying it up at some*

Page

*Distance from the Wound. By Mr.* Edward Ford, *Surgeon of the Westminster General Dispensary.* —— —— 357

VII. *Account of a Calculus extracted from a Cyst in the Neck. By the same.* - 362

VIII. *Observations on the Treatment of Gun-shot Wounds. By* Robert Jackson, *M. D. Physician at Stockton, in the County of Durham.* —— —— 363

IX. *Case of Fracture of the Scull. By Mr.* George Wilkinson, *Surgeon at Sunderland.* 370

X. *Some Account of a Disease lately observed in Infants. By* Thomas Denman, *M. D. Licentiate in Midwifery of the Royal College of Physicians, London.* —— 374

XI. *An Account of two Cases of retroverted Uterus. By Mr.* Richard Croft, *Surgeon in London.* —— —— 380

XII. *An Account of the successful Application of Electricity in a Case of wry Neck. By* William Gilby, *M. D. Physician to the General Hospital at Birmingham.* —— 385

XIII. *A Case of Phthisis Pulmonalis; with Remarks. By Mr.* Edmund Pitts Gapper, *Surgeon at Mere in Wiltshire.* —— 388

Catalogue *of* Books. —— 398

*Address to the Reader.* —— 400

General Index. —— 402

